# Cancer risk among 21st century blood transfusion recipients

**DOI:** 10.1093/annonc/mdw555

**Published:** 2016-11-14

**Authors:** T. O. Yang, B. J. Cairns, G. K. Reeves, J. Green, V. Beral, for the Million Women Study collaborators

**Affiliations:** Cancer Epidemiology Unit, Nuffield Department of Population Health, University of Oxford, Oxford, UK

**Keywords:** blood transfusion, cancer, lymphoma, liver cancer, blood-borne viruses

## Abstract

**Background:**

Some carcinogenic viruses are known to be transmissible by blood transfusion. Intensive viral screening of transfused blood now exists in most countries. In the UK, high-sensitivity nucleic acid amplification tests for hepatitis C virus were introduced in 1999 and it was thought that this would reduce, and possibly eliminate, transfusion-related liver cancer. We aimed to investigate cancer risk in recipients of blood transfusion in 2000 or after.

**Methods:**

A total of 1.3 million UK women recruited in 1998 on average were followed for hospital records of blood transfusion and for cancer registrations. After excluding women with cancer or precancerous conditions before or at the time of transfusion, Cox regression yielded adjusted relative risks of 11 site-specific cancers for women with compared to without prior blood transfusion.

**Results:**

During follow up, 11 274 (0.9%) women had a first recorded transfusion in 2000 or after, and 1648 (14.6%) of them were subsequently diagnosed with cancer, a mean 6.8 years after the transfusion. In the first 5 years after transfusion there were significant excesses for most site-specific cancers examined, presumably because some had preclinical cancer. However, 5 or more years (mean 8 years) after blood transfusion, there were significant excess risks only for liver cancer (adjusted relative risk = 2.63, 95%CI 1.45–4.78) and for non-Hodgkin lymphoma (adjusted relative risk = 1.74, 1.21–2.51). When analyses were restricted to those undergoing hip or knee replacement surgery, the commonest procedure associated with transfusion, these relative risks were not materially altered.

**Conclusions:**

In a large cohort of UK women, transfusions in the 21st century were associated with long-term increased risks of liver cancer and non-Hodgkin lymphoma. Some of these malignancies may have been caused by carcinogenic agents that are not currently screened for in transfused blood.

## Introduction

Some carcinogenic infectious agents are known to be transmissible by blood transfusion. Although screening for hepatitis B and C viruses, human immunodeficiency viruses (HIVs), and human T-cell lymphotrophic viruses (HTLVs) in blood products was introduced in most developed countries during the 1980s and 1990s, transmission of carcinogenic viruses might still have been happening then [1, 2]. To reduce further the risk of virus transmission, nucleic acid amplification testing for Hepatitis C virus was introduced in the late 1990s as a highly sensitive blood screening tool in many countries, including the UK in 1999 [1]. There are no population-based data on cancer risks associated with transfusions in the 21st century, i.e. after the introduction of the nucleic acid amplification test for hepatitis C viruses in the donated blood. Our aim here is to examine on cancer incidence associated with blood transfusions administered in 2000 or later in a large cohort of UK women, among whom we could exclude those with cancer or precancerous conditions (e.g. hepatitis) at the time of or before the transfusion, and among whom we could adjust for known risk factors for cancer, including smoking and alcohol consumption. We investigated transfusion-associated site-specific cancer risks for the 10 most common cancer sites in the cohort, and also for liver cancer, as increased risks of liver cancer before the 21st century blood transfusion have been consistently reported [1, 2].

## Methods

The Million Women Study, as described previously [3], is a cohort of 1.3 million UK women recruited at median age 56 years (interquartile range 52–60 years) through the UK National Breast Screening Programme in 1998 on average. At recruitment, participants provided informed consent and completed questionnaires reporting on socio-demographic and behavioural factors, and about their health. Individual data on hospital admissions and incident cancers in participants was obtained by record linkage to National Health Service (NHS) hospital admission, cancer registry, and death data. Conditions were coded using the International Classification of Diseases, 10th revision (ICD-10) and interventions and procedures, including blood transfusions, were coded using the Office of Population Censuses and Surveys Classification of Interventions and Procedures version 4 (OPCS-4; http://www.hscic.gov.uk/). We used OPCS-4 codes X30-34 to define blood transfusion. The study was approved by the Multi-Centre Research Ethics Committee for Anglia and Oxford.

### Statistical analysis

Follow-up began on 1 January 2000 and the last date of follow-up was 31 December 2013, date of any cancer registration, date of death, or loss to follow up (whichever was earliest). We investigated 11 cancer sites, 10 of which were the most common, having 35 or more cases after blood transfusion, and also liver cancer, as there is strong prior evidence of its association with transfusion [1, 2]. ICD-10 codes used to define each of the 11 cancer sites are given in Supplementary Table S1 in the Appendix, available at *Annals of Oncology* online. Information on hospital admissions, including day cases and overnight stays, was available up to 31 March 2011 (England) or to 31 December 2008 (Scotland). In the UK, transfusions are given as a day-case admission or during an inpatient stay.

Cox regression was used to estimate relative risks (RRs) and 95% confidence intervals (95% CIs) for incident cancer by time since the first recorded blood transfusion (0–4 and 5 or more years after first transfusion) as a time-varying variable, compared with person-years without a prior blood transfusion. We estimated cancer risk by two time periods since transfusion because bleeding and/or chronic anaemia could be a symptom of preclinical cancer, and so the risk of cancer is expected to be high in the first few years after blood transfusion. It seems unlikely, however, that any preclinical cancer causing symptoms that would necessitate a blood transfusion would remain undiagnosed for 5 years or longer.

In the analyses of liver cancer risk, we excluded blood transfusion recipients who had prior or concurrent viral hepatitis, chronic hepatitis, or gastroesophageal varices (ICD-10 codes B1, I85, I86.4, I98.2, K73, and K74). In the analyses of non-Hodgkin lymphoma, myeloma, and leukaemia, we excluded blood transfusion recipients who had prior or concurrent polycythaemia vera, myelodysplastic syndromes, neoplasm of uncertain behaviour of haematological malignancies or unspecific sites, or aplastic anaemia (ICD-10 codes: D45-48 and D60-61). In analyses for endometrial cancer, we excluded women who reported at recruitment that they had a history of hysterectomy. In analyses for ovarian cancer, we excluded women who reported at recruitment that they had a history of bilateral oophorectomy.

In all analyses, age was used as the underlying time variable, and hazard ratios (referred to as relative risks hereafter) were further stratified by year of birth (before 1940, 1940–1945, 1946, and after), region (Scotland and nine regions in England), and socioeconomic status (Townsend social deprivation [4], in quintiles), and adjusted for adult height (<155, 155–159, 160–164, 165–169, 170–174, and 175+ cm), smoking status (never, former, current <15, 15–24, 25+ cigarettes/day), body mass index (<22.5, 22.5–24.9, 25.0–27.4, 27.5–29.9, 30.0–32.4, 32.5–34.9, 35.0–37.4, and 37.5+ kg/m^2^, calculated from reported height and weight), and alcohol consumption (0, 1–4, 5–14, and 15+ g/day) [5]. Missing values (<6%) were treated as a separate category. We present standard *p* values and 95% confidence intervals, but only considered the *P* values statistically significant after correction for multiple comparisons using the Holm–Bonferroni method [6] at *P *<* *0.05. To compare cancer risk before and after full adjustment, we calculated the percentage attenuation of the minimally adjusted RR after fully adjustment additionally for adult height, smoking status, body mass index, and alcohol consumption, i.e. (minimally adjusted RR−fully adjusted RR)/(minimally adjusted RR−1).

We also estimated the excess absolute cancer risks per 1000 women in the 5–9 years after transfusion. These were estimated by logistic models using STATA 14.2 (*xtgee* and *margins*), adjusted for age (in 5 year spans) and the same above-mentioned covariates including year of birth, region, socioeconomic status, adult height, smoking status, body mass index, and alcohol consumption.

## Results

Among 1 299 246 women without prior cancer followed for 16.5 million person-years after 1 January 2000 (12.7 years per women on average), 11 274 (0.9%) women had one or more blood transfusions with the first one recorded in 2000 or later. In 2000–2013, 160 041 cancers occurred in the entire cohort, of which 1648 were in the 11 274 women who had had a blood transfusion in 2000 or later. The characteristics of women with and without a record of blood transfusion in 2000 or later are shown in Table 1. Compared with women without a record of blood transfusion, women with a record of blood transfusion were older at baseline, more likely to come from the most deprived tertile of the population, to be a current smoker, to have greater body mass index, and to drink less alcohol.

**Table 1:** Characteristics of women with and without a record of blood transfusion, censored at any cancer diagnosis

	Women without a record of blood transfusion	Women with a record of blood transfusion
	*n*=1 287 972	*n*=11 274
Age at start of follow up in years (mean, SD)	58 (5)	60 (5)
Information taken at recruitment		
Lowest quintile of socioeconomic status (%)	20	25
Have ever smoked (%)	49	54
Body mass index in kg/m^2^ (mean, SD)	26 (5)	27 (6)
Alcohol consumption in g/day (mean, SD)	7 (9)	6 (9)
Number of cancer cases (ICD-10 code)^a^		
Colorectal cancer (C18-20)	17 547	291
Liver cancer (C22)	1227	25
Pancreatic cancer (C25)	4135	54
Lung cancer (C34)	16 587	197
Breast cancer (C50)	55 284	265
Endometrial cancer (C54)	9977	40
Ovarian cancer (C56)	7887	55
Renal cancer (C64)	3234	48
Non-Hodgkin lymphoma (C82-85)	6099	114
Myeloma (C90)	2424	38
Leukaemia (C91–93, 95)	2762	70
Other cancers	31230	451

a ICD-10, International Classification of Diseases, Revision 10.

**Figure 1.**
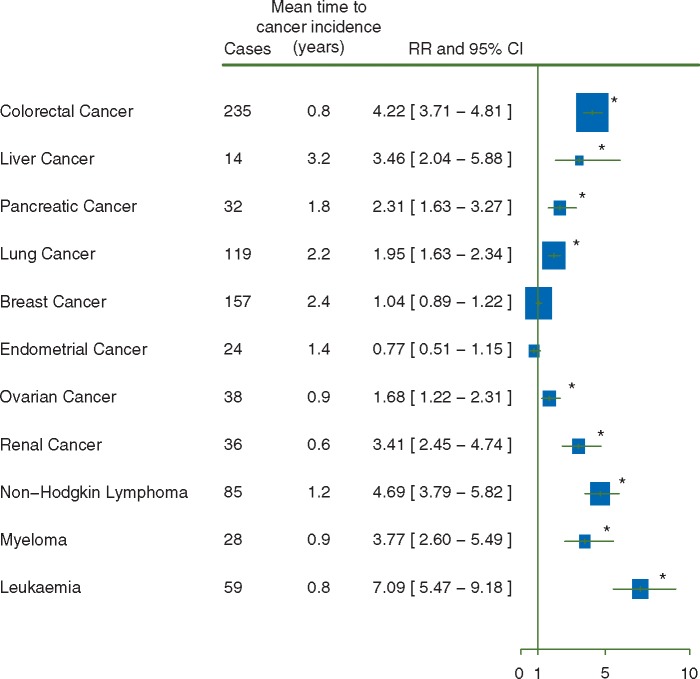
Risk of cancer in the first 5 years after the 21st century blood transfusion. (♣) Risks were adjusted for age, year of birth, region, socioeconomic status, height, body mass index, smoking, and alcohol consumption. *P<0.05 after correction for multiple comparisons.

Fully adjusted relative risks for the 10 most common cancers and liver cancer in the first 5 years after transfusion, i.e. after adjustment for age, year of birth, region, socioeconomic status, adult height, body mass index, smoking, and alcohol consumption, are shown in Figure 1. Results for minimally adjusted RRs and RRs with additional adjustment for each factor can be found in the Supplementary Appendix, available at *Annals of Oncology* online. Relative risks of most cancers were relatively insensitive to additional adjustment (change of relative risk <15% after adjustment), but the relative risk of lung cancer was sensitive to adjustment for smoking status (25% change after adjustment), and the relative risk of endometrial cancer was sensitive to adjustment for body mass index (35% change after adjustment).

**Figure 2.**
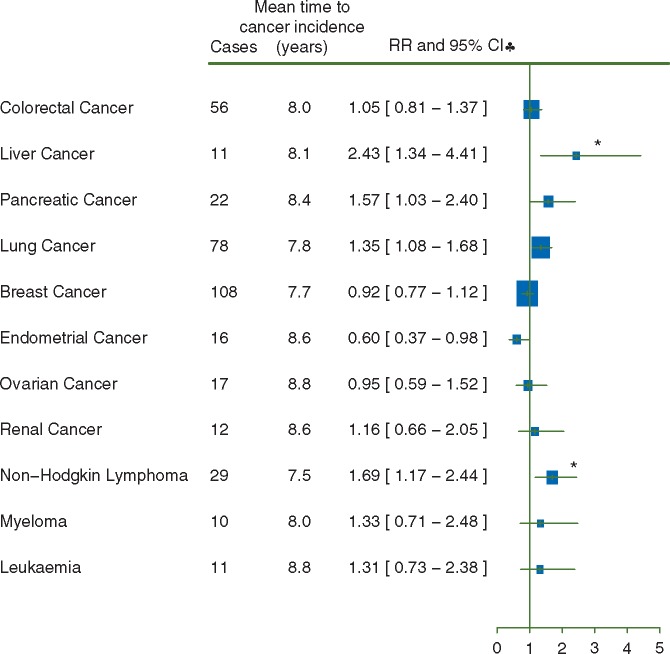
Risk of cancer 5 or more years after the 21st century blood transfusion. (♣) Risks were adjusted for age, year of birth, region, socioeconomic status, height, body mass index, smoking, and alcohol consumption. *P<0.05 after correction for multiple comparisons.

For 9 out of 11 cancer sites RRs were increased in the first 5 years after blood transfusion (Figure 1, *P *<* *0.05 after correction for multiple comparisons), as expected, because some of the transfusions could well have been for precancerous conditions. Risks were much lower in the period 5 or more years after transfusion than in the period less than 5 years after transfusion (Figure 2; mean time of cancer incidence since transfusion = 8.0 years). The only RRs that remained significantly elevated 5 or more years after transfusion, after allowing for multiple comparisons, were those for liver cancer (RR = 2.43, 95%CI 1.34–4.41, *P *=* *0.04) and for non-Hodgkin lymphoma (RR = 1.69, 95%CI 1.14–2.44, *P *=* *0.05). Transfusion-associated RRs for liver cancer and non-Hodgkin lymphoma were slightly attenuated by adjustment for smoking, alcohol consumption, and body mass index (risk attenuation 13% for liver cancer and 7% for non-Hodgkin lymphoma). Sensitivity analysis excluding women with missing values for any of the adjustment variables yielded similar results for liver cancer (RR = 2.48, 95%CI 1.33–4.65) and non-Hodgkin lymphoma (RR = 1.79, 95%CI 1.23–2.60).

Table 2 shows the conditions and procedures recorded at the time of transfusion in the 11 women who developed liver cancer and the 29 women who had non-Hodgkin lymphoma 5 or more years after blood transfusion. Of the 11 women who developed liver cancer, about half (55%, 6/11) had had surgery at the time of transfusion (of which half, 3, were for hip or knee replacements) and 3 had investigations for non-cancer related, non-variceal gastrointestinal bleeding. None had a condition associated with liver disease or with liver cancer (having excluded all women with prior viral hepatitis, chronic hepatitis, or gastroesophageal varices in the analyses). Relative risk for 5 or more years after transfusion was similar when we restricted analysis to transfusion during hospital admissions for hip or knee replacement (three liver cancers, RR = 2.10, 95%CI 0.68–6.55), although the confidence interval was wide.

**Table 2:** Procedures and conditions recorded at the time of transfusion in women who developed liver cancer or non-Hodgkin lymphoma 5 or more years after blood transfusion

Conditions and procedures recorded at the time of transfusion
Liver cancer (11 in total) occurring 5 or more years after blood transfusion:
6 had a transfusion associated with a surgical procedure, including 3 with knee or hip replacement surgery
3 had a transfusion associated with investigations for gastrointestinal bleeding (not varices nor cancer)
2 had transfusion for other conditions, none associated with liver disease or cancer

Non-Hodgkin lymphoma (29 in total) occurring 5 or more years after blood transfusion:
16 had a transfusion associated with a surgical procedure, including 12 with knee or hip replacement surgery
9 had a transfusion associated with investigations for gastrointestinal bleeding (not varices nor cancer)
4 had transfusion for other conditions, none associated with non-Hodgkin lymphoma

Of the 29 women who developed non-Hodgkin lymphoma 5 or more years after transfusion, about half (55%, 16/29) had surgery at the time of transfusion (of which three quarters, 12, were for hip or knee replacements) and 9 had investigations for non-cancer related, non-variceal gastrointestinal bleeding. The remaining four women had a variety of conditions not associated with non-Hodgkin lymphoma. All women with prior polycythaemia vera, myelodysplastic syndromes, neoplasm of uncertain behaviour of haematological malignancies or unspecific sites, and aplastic anaemia were excluded from the analyses. Because non-Hodgkin lymphoma is weakly associated with a wide range of haematological conditions and other chronic conditions [7, 8], we further excluded women with a range of chronic conditions including rheumatologic diseases, other anaemias, viral hepatitis, and renal failure at baseline, and there was no substantial change in the relative risk after these further exclusions (RR for 5 or more years after transfusion 2.05, 95%CI 1.21–3.47, 14 cases). Risk was similar when analysis was restricted to transfusions associated with hip or knee replacements (12 non-Hodgkin lymphomas, RR = 2.14, 95%CI 1.21–3.77).

We estimated the absolute cancer incidence per 1000 person-years for liver cancer and non-Hodgkin lymphoma in the 5–9 years after blood transfusion, after adjustment. The absolute risk for liver cancer was 0.16 (95%CI 0.08–0.24) per 1000 person-years 5 or more years after blood transfusion, compared with 0.07 (95%CI 0.06–0.07) per 1000 person-years among women without a history of blood transfusion who had the same lifestyle profile. This is equivalent to about 1 excess liver cancer in every 2300 blood recipients 5–9 years after transfusion. For non-Hodgkin lymphoma, the absolute risks were 0.40 (95%CI 0.31–0.49) per 1000 person-years 5 or more years after blood transfusion compared to 0.28 (95%CI 0.27–0.29) per 1000 person-years among women without a history of blood transfusion, equivalent to about 1 excess non-Hodgkin lymphoma in every 1700 blood recipients 5–9 years after transfusion. For both cancers combined, there was about 1 excess cancer in every 1000 blood recipients in the 5–9 years after transfusion.

## Discussion

In this report, we investigated risk of the 10 commonest cancers and of liver cancer in the 21st century blood transfusion recipients, an era after the introduction of nucleic acid amplification testing for hepatitis C virus, a more sensitive test than the previous antibody-based tests [1]. We found significant excesses both for liver cancer and for non-Hodgkin lymphoma 5 or more years (mean 8 years) after first known blood transfusion. The reasons the transfusions were done in these women appeared to be for conditions unlikely to be pre-clinical manifestations of either liver cancer or non-Hodgkin lymphoma; for example, a third of the transfusions were associated with hip or knee replacement surgery. In the analyses, we excluded women with known possible precancerous conditions at or before transfusion (as well as women without known cancer) and adjusted for potential confounding factors, such as smoking or alcohol consumption.

Cancers that are known to be caused by viruses are hepatocellular carcinoma (hepatitis viruses), some lymphomas (Epstein–Barr virus), nasopharyngeal carcinoma (Epstein–Barr virus), anogenital cancers and head and neck cancers (Human Papillomavirus), adult T-cell leukaemia (HTLV), and Kaposi sarcoma (Kaposi sarcoma-associated herpesvirus) [9]. Screening for hepatitis viruses, HTLV, and HIV in blood is a policy in UK and most developed countries. Since the introduction of nucleic acid amplification testing for hepatitis C virus in 1999 in the UK, it has been reported that only about 1 in a million blood transfusions carry the virus, and no known case of transfusion-related viral hepatitis soon after transfusion has been reported in the UK since 2005 [10]. However, cancers and other conditions long after transfusion have been less studied.

No other study has reported on cancer risk associated with the 21st century blood transfusion. A large study from Denmark and Sweden reported relative risks 5–9 years after blood transfusion in 1992–2002 of 1.86 for liver cancer and 1.20 for non-Hodgkin lymphoma [2]. The authors were not able to exclude people with prior precancerous conditions, and did not have information on life-style risk factors, and concluded that they could not exclude the possibility of confounding by these factors. The RRs found here for liver cancer and for non-Hodgkin lymphoma were significantly increased even after adjusting for known risk factors for cancer, including smoking, alcohol and body mass index and excluding women with possible precancerous conditions. Furthermore, the relative risks in this study did not change substantially in sensitivity analyses restricted to transfusions associated with hip or knee replacement surgery, where confounding by the indication for transfusion seems unlikely.

Given that confounding by known risk factors or indications for transfusion seem unlikely, the possibility that the excess risks of liver cancer and of non-Hodgkin lymphoma many years after the 21st century transfusions may be due to carcinogenic agents being transmitted in transfused blood needs to be considered. For liver cancer the extremely low carrier rate of hepatitis B and hepatitis C viruses (<0.01%) reported among UK donors [11, 12] and the low false-negative rate in blood screening (<1%) has been estimated to correspond to a risk of less than 5 per million blood recipients under nucleic acid amplification tests [1]. However, we found about 1 excess liver cancer in every 2300 blood recipients in the 5–9 years after transfusion, much higher than the reported prevalence of the known hepatitis viruses. Breakthrough infections of screened viruses are unlikely to explain the excess of liver cancer, and the role of other viruses associated with liver cancer that were not screened for, such as Hepatitis G virus [13], needs to be investigated.

The excess risk of transfusion-associated non-Hodgkin lymphoma has been consistently reported previously [14–28]. Some have proposed the possibility of introduction of donor lymphoma cells through blood, although a large register-based study of cancer in the donors did not support this hypothesis [29]. Others have suggested that chemical or biological components of transfused blood may cause a temporary immunomodulation [30, 31], promoting lymphoma development, but this hypothesis would not explain why risks remain high many years after transfusion. Emerging evidence has suggested possible associations between viruses and some non-Hodgkin lymphoma cases, including Epstein–Barr virus [32] and again Hepatitis G virus [33, 34], and these viruses are not screened for in transfused blood.

Our findings are limited by the type of information on transfusion that was available. We did not have information on the type or the number of transfusions, nor on transfusions done before the hospital records began or on unrecorded transfusions. We could not investigate transfusion-associated cancer risks longer than 10 years after transfusion, because we restricted our study to the transfusions in the 21st century. Additionally, associations may be confounded by unmeasured factors, including conditions not recorded at the time of transfusion, or by transfusion history or other history prior to the period of data availability. Furthermore, we have no information for men or people transfused at younger ages.

Blood transfusions are generally given for life-threatening situations, and the immediate benefits would out-weight the long-term risk of cancer. Nevertheless, the long-term excess risks of liver cancer and of non-Hodgkin lymphoma warrants search for carcinogenic agents not currently screened for in transfused blood.

Key message

Screening for the blood-borne viruses in transfused blood has been extensive in the UK during the 21st century. In this large prospective study of UK women, we found increased risks of liver cancer and non-Hodgkin lymphoma more than 5 years after transfusion, suggesting that some of these malignancies may be caused by carcinogenic agents that are not currently screened for in transfused blood.

## Supplementary Material

Supplementary Data
