# Supplementary material for: Cancer risk among 21st century blood transfusion recipients
Source: Ann Oncol. 2016 Nov 14;28(2):393–9. doi: 10.1093/annonc/mdw555 (PMC5391695; doi:10.1093/annonc/mdw555)
Supplement: Supplementary Data [file mdw555_supplementary_data.zip › Appendix_20160416Owen.pdf]

Cancer risk among 21st century blood transfusion recipients

T. O. Yang[1], B. J. Cairns[1], G. K. Reeves[1], J. Green[1], and V. Beral[1]

for the Million Women Study Collaborators

[1] Cancer Epidemiology Unit, Nuffield Department of Population Health, University of Oxford, Oxford, United Kingdom

[1] Cancer Epidemiology Unit, Nuffield Department of Population Health, University of Oxford, Oxford, United Kingdom

Appendix

Page 2 Million Women Study Collaborators

Page 3-5 Table: Unadjusted and adjusted risks of 11 site-specific cancers by time since blood transfusion

The Million Women Study Collaborators are:

Hayley Abbiss, Simon Abbott, Rupert Alison, Naomi Allen, Miranda Armstrong, Krys Baker, Angela Balkwill, Emily Banks, Isobel Barnes, Valerie Beral, Judith Black, Roger Blanks, Kathryn Bradbury, Anna Brown, Benjamin Cairns, Karen Canfell, Dexter Canoy, Andrew Chadwick, Francesca Crowe, Dave Ewart, Sarah Ewart, Lee Fletcher, Sarah Floud, Toral Gathani, Laura Gerrard, Adrian Goodill, Jane Green, Lynden Guiver, Michal Hozak, Isobel Lingard, Sau Wan Kan, Nicky Langston, Bette Liu, Kath Moser, Kirstin Pirie, Gillian Reeves, Keith Shaw, Emma Sherman, Helena Strange, Sian Sweetland, Sarah Tipper, Ruth Travis, Lyndsey Trickett, Lucy Wright, Owen Yang, Heather Young.

Appendix: Unadjusted and adjusted risks of 11 cancers by time since blood transfusion

		Years since blood transfusion			
		1-4 years		5 or more years	
		n	RR (95%CI)	n	RR (95%CI)
Colorectal Cancer (C18-20)	Adjusted for age, region, socioeconomic status, and year of birth	235	4.28 (3.76 - 4.87)	56	1.07 (0.82 - 1.39)
	Additionally adjusted for…				
	Adult height		4.30 (3.78 - 4.89)		1.07 (0.82 - 1.39)
	Body mass index		4.26 (3.74 - 4.84)		1.06 (0.82 - 1.38)
	Smoking status		4.25 (3.73 - 4.83)		1.06 (0.82 - 1.38)
	Alcohol consumption		4.28 (3.76 - 4.87)		1.07 (0.82 - 1.39)
	**All above**		**4.22 (3.71 - 4.81)**		**1.05 (0.81 - 1.37)**
			χ_1^2 = 1.89 ***		χ_1^2 < 0.01
Liver Cancer (C22)	Adjusted for age, region, socioeconomic status, and year of birth	14	3.81 (2.25 - 6.46)	11	2.63 (1.45 - 4.78)
	Additionally adjusted for…				
	Adult height		3.79 (2.24 - 6.43)		2.63 (1.45 - 4.77)
	Body mass index		3.60 (2.12 - 6.10)		2.49 (1.37 - 4.53)
	Smoking status		3.74 (2.20 - 6.34)		2.60 (1.43 - 4.71)
	Alcohol consumption		3.71 (2.19 - 6.30)		2.59 (1.42 - 4.69)
	**All above**		**3.46 (2.04 - 5.88)**		**2.43 (1.34 - 4.41)**
			χ_1^2 = 0.95 ***		χ_1^2 = 0.34 *
Pancreatic Cancer (C25)	Adjusted for age, region, socioeconomic status, and year of birth	32	2.43 (1.72 - 3.45)	22	1.65 (1.08 - 2.51)
	Additionally adjusted for…				
	Adult height		2.43 (1.71 - 3.44)		1.65 (1.08 - 2.51)
	Body mass index		2.39 (1.69 - 3.38)		1.62 (1.06 - 2.46)
	Smoking status		2.36 (1.67 - 3.35)		1.61 (1.06 - 2.46)
	Alcohol consumption		2.41 (1.70 - 3.41)		1.64 (1.07 - 2.49)
	**All above**		**2.31 (1.63 - 3.27)**		**1.57 (1.03 - 2.40)**
			χ_1^2 = 0.43 ***		χ_1^2 = 0.06
Lung Cancer (C34)	Adjusted for age, region, socioeconomic status, and year of birth	119	2.20 (1.84 - 2.64)	78	1.48 (1.18 - 1.85)
	Additionally adjusted for…				
	Adult height		2.20 (1.83 - 2.63)		1.48 (1.18 - 1.85)
	Body mass index		2.26 (1.89 - 2.71)		1.52 (1.22 - 1.90)
	Smoking status		1.98 (1.65 - 2.37)		1.36 (1.09 - 1.70)
	Alcohol consumption		2.11 (1.76 - 2.53)		1.43 (1.14 - 1.79)
	**All above**		**1.95 (1.63 - 2.34)**		**1.35 (1.08 - 1.68)**
			χ_1^2 = 0.33 ***		χ_1^2 = 0.03

After correction for multiple comparisons

* p<0.05 and >=0.01
** p<0.01 and >=0.001
*** p<0.001

		Years since blood transfusion			
		1-4 years		5 or more years	
		n	RR (95%CI)	n	RR (95%CI)
Breast Cancer (C50)	Adjusted for age, region, socioeconomic status, and year of birth	157	1.05 (0.90 - 1.23)	108	0.93 (0.77 - 1.12)
	Additionally adjusted for…				
	Adult height		1.05 (0.90 - 1.23)		0.93 (0.77 - 1.13)
	Body mass index		1.04 (0.89 - 1.21)		0.92 (0.76 - 1.11)
	Smoking status		1.05 (0.89 - 1.22)		0.93 (0.77 - 1.12)
	Alcohol consumption		1.06 (0.91 - 1.24)		0.94 (0.78 - 1.13)
	**All above**		**1.04 (0.89 - 1.22)**		**0.92 (0.77 - 1.12)**
			χ_1^2 < 0.01		χ_1^2 = 0.03
Endometrial Cancer (C54)	Adjusted for age, region, socioeconomic status, and year of birth	24	0.91 (0.61 - 1.36)	16	0.71 (0.43 - 1.15)
	Additionally adjusted for…				
	Adult height		0.91 (0.61 - 1.36)		0.71 (0.43 - 1.15)
	Body mass index		0.77 (0.51 - 1.14)		0.60 (0.37 - 0.98)
	Smoking status		0.92 (0.62 - 1.38)		0.71 (0.44 - 1.16)
	Alcohol consumption		0.89 (0.60 - 1.33)		0.69 (0.42 - 1.13)
	**All above**		**0.77 (0.51 - 1.15)**		**0.60 (0.37 - 0.98)**
			χ_1^2 = 0.22		χ_1^2 = 0.58
Ovarian Cancer (C56)	Adjusted for age, region, socioeconomic status, and year of birth	38	1.70 (1.23 - 2.33)	17	0.95 (0.59 - 1.54)
	Additionally adjusted for…				
	Adult height		1.70 (1.24 - 2.34)		0.96 (0.59 - 1.54)
	Body mass index		1.68 (1.22 - 2.32)		0.95 (0.59 - 1.53)
	Smoking status		1.70 (1.23 - 2.33)		0.95 (0.59 - 1.54)
	Alcohol consumption		1.69 (1.23 - 2.33)		0.95 (0.59 - 1.53)
	**All above**		**1.68 (1.22 - 2.31)**		**0.95 (0.59 - 1.52)**
			χ_1^2 = 0.13 **		χ_1^2 = 0.09
Renal Cancer (C64)	Adjusted for age, region, socioeconomic status, and year of birth	36	3.75 (2.70 - 5.21)	12	1.27 (0.72 - 2.24)
	Additionally adjusted for…				
	Adult height		3.76 (2.71 - 5.23)		1.27 (0.72 - 2.25)
	Body mass index		3.51 (2.53 - 4.88)		1.19 (0.67 - 2.10)
	Smoking status		3.69 (2.65 - 5.13)		1.26 (0.71 - 2.22)
	Alcohol consumption		3.67 (2.64 - 5.10)		1.24 (0.71 - 2.20)
	**All above**		**3.41 (2.45 - 4.74)**		**1.16 (0.66 - 2.05)**
			χ_1^2 = 1.12 ***		χ_1^2 = 0.02

After correction for multiple comparisons

* p<0.05 and >=0.01
** p<0.01 and >=0.001
*** p<0.001

Appendix p4

		Years since blood transfusion			
		**1-4 years**		**5 or more years**	
		**n**	**RR (95%CI)**	**n**	**RR (95%CI)**
Non-Hodgkin Lymphoma (C82-85)	Adjusted for age, region, socioeconomic status, and year of birth	85	4.86 (3.92 - 6.02)	29	1.74 (1.21 - 2.51)
	Additionally adjusted for…				
	Adult height		4.88 (3.93 - 6.05)		1.75 (1.21 - 2.52)
	Body mass index		4.75 (3.83 - 5.89)		1.71 (1.18 - 2.46)
	Smoking status		4.83 (3.90 - 5.99)		1.74 (1.20 - 2.50)
	Alcohol consumption		4.81 (3.88 - 5.96)		1.73 (1.20 - 2.49)
	**All above**		**4.69 (3.79 - 5.82)**		**1.69 (1.17 - 2.44)**
			χ^2_1 = 2.06 ***		χ^2_1 = 0.11 *
Myeloma (C90)	Adjusted for age, region, socioeconomic status, and year of birth	28	3.86 (2.65 - 5.61)	10	1.36 (0.73 - 2.54)
	Additionally adjusted for…				
	Adult height		3.86 (2.65 - 5.61)		1.36 (0.73 - 2.53)
	Body mass index		3.78 (2.60 - 5.49)		1.34 (0.72 - 2.49)
	Smoking status		3.87 (2.67 - 5.63)		1.37 (0.73 - 2.55)
	Alcohol consumption		3.84 (2.64 - 5.58)		1.35 (0.73 - 2.52)
	**All above**		**3.77 (2.60 - 5.49)**		**1.33 (0.71 - 2.48)**
			χ^2_1 = 1.29 ***		χ^2_1 < 0.01
Leukaemia (C91-93, 95)	Adjusted for age, region, socioeconomic status, and year of birth	59	7.32 (5.65 - 9.49)	11	1.36 (0.75 - 2.47)
	Additionally adjusted for…				
	Adult height		7.36 (5.68 - 9.53)		1.37 (0.75 - 2.48)
	Body mass index		7.18 (5.54 - 9.31)		1.33 (0.74 - 2.41)
	Smoking status		7.27 (5.61 - 9.43)		1.35 (0.75 - 2.45)
	Alcohol consumption		7.25 (5.59 - 9.39)		1.35 (0.74 - 2.44)
	**All above**		**7.09 (5.47 - 9.18)**		**1.31 (0.73 - 2.38)**
			χ^2_1 = 3.33 ***		χ^2_1 < 0.01

After correction for multiple comparisons

* p<0.05 and >=0.01
** p<0.01 and >=0.001
*** p<0.001
